# The Relational Experience of Fear of Cancer Recurrence in Family Caregivers: A Reflexive Thematic Analysis Study

**DOI:** 10.3390/curroncol32040209

**Published:** 2025-04-01

**Authors:** Jani Lamarche, Faye Ajmera, Jonathan Avery, Ghizlène Sehabi, Sophie Lebel, Rinat Nissim

**Affiliations:** 1 School of Psychology, University of Ottawa, 136 Jean-Jacques-Lussier Private, Ottawa, ON K1N 6N5, Canada; gseha100@uottawa.ca (G.S.); sophie.lebel@uottawa.ca (S.L.); 2 Department of Supportive Care, Princess Margaret Cancer Centre, University Health Network, Toronto, ON M5G 2M9, Canada; faye.ajmera@mail.utoronto.ca (F.A.); rinat.nissim@uhn.ca (R.N.); 3 Department of Supportive Care, BC Cancer, Vancouver, BC V5Z 4E6, Canada; jonathan.avery@bccancer.bc.ca; 4 Department of Psychiatry, Temerity Faculty of Medicine, University of Toronto, 250 College Street, Toronto, ON M5T 1R8, Canada

**Keywords:** family caregivers, fear of cancer recurrence, FCR, cancer, focus group, interviews, qualitative, oncology, survivorship

## Abstract

Fear of cancer recurrence (FCR) affects approximately 50% of family caregivers. While FCR in cancer patients has been well-documented, less is known about the experience of FCR in family caregivers. This study aimed to qualitatively explore the distinct characteristics of FCR in family caregivers. A focus group and semi-structured interviews were conducted via videoconferencing with family caregivers of cancer survivors (stages I–III, finished treatment, no recurrence). Participants were recruited through Canadian hospitals, community partners, and social media. The focus group and qualitative interviews explored family caregivers’ experiences of FCR, including its content, frequency, impact, and management. A reflexive thematic analysis was used. In total, twenty family caregivers participated. Six participated in the focus group. Sixteen participated in the interviews. Two participated in both. Family caregivers described their experience of FCR as all-consuming, constant, and marked by a sense of helplessness. Qualitative analysis revealed a major theme of relational aspects of FCR in family caregivers, with the following four inter-related themes: patient-centric hypervigilance, self-silencing, FCR as isolating, and finding support. This qualitative study examined the experiences of family caregivers living with FCR. Our findings highlight that relational factors shape how family caregivers experience and manage their FCR. High-quality survivorship care should be redefined to include FCR interventions tailored to family caregivers.

## 1. Introduction

Fear of cancer recurrence (FCR), defined as the “fear, worry or concern that cancer may come back or progress” [1], is a prevalent and distressing experience among individuals who have completed their cancer treatments [2]. FCR represents the primary unmet need of survivors post-treatment [3], affecting an estimated 59% [2], and is associated with impaired functioning, psychological distress, a lower quality of life (QoL) [2,3,4,5], and increased costs for the medical system (e.g., accrued outpatient and emergency department visits) [6,7,8]. Importantly, there is evidence from two meta-analyses [9,10] that group and individual interventions, such as Fear Of Recurrence Therapy (FORT) [11], can successfully reduce clinical FCR among survivors.

While FCR has been extensively studied in cancer survivors [2,3,4,5,12], there is a growing recognition of the unique challenges faced by family caregivers in managing their own fears and anxieties related to the possibility of their loved ones’ cancer recurring.Family caregivers, who provide unpaid support, play a crucial role in the treatment and care of individuals diagnosed with cancer [13]. Systematic reviews have identified that approximately 50% of family caregivers experience equal or greater levels of FCR than survivors themselves [14,15,16]. Significant correlations, comparable to those observed in cancer survivors, have also been found between FCR and poorer psychosocial outcomes (e.g., depression and anxiety) in family caregivers [16]. Furthermore, there is evidence suggesting that the FCR levels in family caregivers can influence those experienced by survivors [17]. Therefore, treating FCR in family caregivers could potentially improve outcomes for both family caregivers and survivors. While age, gender, treatment type, and illness perception (i.e., understanding of illness and beliefs about consequences and causes) have been identified as potential risk factors [14,15,16], there remains much to be understood about the distinct characteristics of family caregivers’ experiences of FCR. Although conceptual models are emerging [18,19], a great deal of our understanding still derives from the experiences of cancer survivors [1,2,3,12,20,21,22].

Qualitative explorations of caregivers’ fears related to cancer recurrence are beginning to appear. Two recent qualitative articles [23,24] by an Australian research team identified themes that mirror the experiences of survivors while highlighting some that are unique to family caregivers. Among the themes resembling those articulated by survivors [12], fear and uncertainty (including intrusive/pervasive thoughts, triggers, and hypervigilance), hopelessness, and liminality (i.e., difficulties planning for the future) were identified [23,24]. Distinct family caregiver themes included concerns about losing their loved ones or witnessing their suffering, the caregiver’s role as a protector, and feeling unprepared [23,24].

While these qualitative studies by the Australian group [23,24] included family caregivers of those with advanced cancer, our study focuses on family caregivers of survivors with stage I–III cancer (who have completed their cancer treatment with no recurrence). The goal of this present study is to understand how family caregivers describe the experience of FCR and identify their unique needs in relation to FCR. Understanding the experience of FCR in family caregivers is essential for the development of tailored caregiver-specific models, measures, interventions, and support services.

## 2. Materials and Methods

### 2.1. Study Design

This qualitative study used the combination of a focus group and semi-structured interviews to explore the experiences of FCR in family caregivers between March 2022 and December 2023. Initially designed as a focus group study, we pivoted to include interviews due to recruitment challenges related to the focus group during the COVID-19 pandemic. Reflexive thematic analysis [25] was used to analyze the data.

Ethics approval was obtained from the University of Ottawa (H-05-20-5584), The Ottawa Hospital (20230147-01H), and the Princess Margaret Cancer Centre (21-5060.3).

### 2.2. Participants

#### 2.2.1. Focus Group

Family caregivers were recruited through the Princess Margaret Cancer Centre and Cancer Chat Canada, operated by the De Souza Institute, an affiliate of the University Health Network, through advertisements and clinician referrals. We recruited individuals who (1) were 18 years of age or older; (2) self-identified as a family caregiver of an adult cancer patient (any type of cancer, stages I–III) seen at the Princess Margaret Cancer Centre, who had completed their treatments (with the exception of adjuvant chemotherapy or hormone therapy) and had not had a recurrence of their cancer; (3) experienced cancer-related fears; and (4) were interested in and able to attend a virtual focus group to discuss coping with cancer-related fears. The exclusion criteria included (1) family caregivers of a pediatric cancer survivor and (2) non-English speakers.

#### 2.2.2. Semi-Structured Interviews

The semi-structured interviews included in this study were conducted as part of a larger study evaluating the acceptability of the newly adapted Family Caregiver-Fear Of Recurrence Therapy (FC-FORT) [26,27,28] addressing FCR in family caregivers and delivered online. Women who were family caregivers caring for an adult cancer survivor (any cancer type, stages I–III, completed treatments, no recurrence), experiencing clinical FCR (a score of ≥13 or greater on the Caregiver Version of the Fear of Cancer Recurrence Inventory-Short Form) [29,30], fluent in English, with a computer and stable internet access, were recruited.

### 2.3. Procedure

#### 2.3.1. Focus Group

The focus group occurred online through the Microsoft Teams platform and was facilitated by the co-authors (RN and JA). Prior research on FCR in family caregivers was used to develop a focus group guide that promoted open discussion, probed for details about family caregivers’ experiences of FCR, and ensured that all participants had an opportunity to share. The focus group guide included questions about the participants’ overall experiences as family caregivers, their experience of FCR (e.g., triggers and thoughts) and related coping strategies, and their needs regarding FCR interventions. In addition, a self-reported demographic questionnaire was completed by each participant via RedCap.

#### 2.3.2. Semi-Structured Interviews

The procedures for the semi-structured interviews are described elsewhere [26,27,28]. In short, the participants took part in a one-on-one interview (30–60 min) via videoconferencing within a few weeks of completing their participation in FC-FORT. The interviews were conducted by JL or a trained research assistant. These interviews focused on the participants’ experience of the FC-FORT [26,27,28] (e.g., impact on FCR, helpful/unhelpful strategies, usability of materials, and group cohesion), but were deemed relevant to the current analysis because the participants often contextualized their answers by speaking about their experiences of FCR in general. The participants were also asked to complete a sociodemographic questionnaire via Qualtrics.

### 2.4. Analysis

The interviews and focus group were audio-recorded, transcribed verbatim, and de-identified. Transcripts were managed using the NVivo12 software. To ensure the relevancy of each interview to the current analysis, FA first reviewed each of the semi-structured interview transcripts and extracted sections pertinent to family caregivers’ experiences of FCR. Reflexive thematic analysis was used [25]. First, the research team independently read the focus group transcript and the interview segments extracted by FA and met to discuss and create initial codes. Then, JL and FA met weekly to analyze the transcripts and discuss discrepancies. Themes related to family caregivers’ experiences of FCR were identified by comparing the initial codes and further developed through a process of inductive coding. The research team met bi-weekly to provide feedback on the coding procedures and review emerging themes. These meetings allowed for a shared and collaborative interpretation of the data, promoted reflection, and limited biases.

## 3. Results

### 3.1. Participant Characteristics 

In total, 20 family caregivers participated in the study (Table 1). Sixteen family caregivers, all identifying as women, participated in the exit interviews, six family caregivers (five women and one man) participated in the focus group, and two family caregivers, both identifying as women, participated in both. The mean age was 51.4 years (range = 21–74). The participants were White (65%), South Asian (10%), Chinese (5%), and Hispanic (5%). The participants provided care to their spouses/partners (45%), adult children (25%), parents (25%), and other family members (5%; Table 2). The family caregivers were mostly married/common law partners (65%), university/college graduates (75%), employed full-time (40%), and earned ≥USD 60,000 annually (50%). The most prevalent types of cancer diagnoses were breast (20%) and prostate (20%).

### 3.2. Qualitative Findings

#### 3.2.1. The Overall Experience of FCR

Our analysis identified that FCR was all-consuming for family caregivers. The participants described FCR as a consistent experience in their lives. Thoughts of recurrence occurred “every single day”, with the belief that this fear would last “forever” and they would “never not be afraid”. Many family caregivers discussed how their experience with fear left them feeling “helpless” and how cancer recurrence was something that they “[couldn’t] control”. One participant discussed the process of trying to rationalize their fear without success, as follows:

“Because if you’re afraid of cancer recurrence in your partner, that’s an emotion. You can’t really deal with that on a cognitive rational level. You can’t talk yourself out of [it], and God knows I have tried.”

[P1]

Consequently, the participants felt “powerless” over their situation. They described how attempts to “deal” with their fear were unsuccessful, resulting in them becoming “stuck” thinking about negative outcomes and being unable to “get out of [their thoughts]”, as follows:

“I can be triggered walking down a grocery aisle. And seeing something in a grocery aisle, it triggers me. I can hear something on the news and be triggered. I could watch a tv show and be triggered. Like the triggering happens everywhere. And I don’t think it’s avoidable unless you commit yourself to a room and just never expose yourself to anything in the outside world. And even that would be triggering because you’d be isolated again the way that I was isolate… I just, I feel like, for the most part, since [date], which is when I got the phone call from my daughter that she had breast cancer, I feel like I’ve been driving around in a car that’s always in fifth gear. Like it’s at its max capacity.”

[P12]

A major theme that was identified in our analysis was the relational aspects of FCR in family caregivers. This relationality is captured in the following four inter-related sub-themes: patient-centric hypervigilance; self-silencing; FCR as isolating; and finding support. Together, these sub-themes not only convey the experience of FCR, but also shed light on the dynamics contributing to its persistent and all-consuming nature.

#### 3.2.2. Patient-Centric Hypervigilance

This constant FCR manifested for the participants in the form of a heightened awareness of the patient’s symptomology, discussing how they are “constantly watching [the patient] for aches and pains, signs and symptoms of anything going on”. As a result, the participants mentioned how their FCR was triggered by occurrences that would otherwise be considered normal or commonplace, as follows:

“She says she has a headache… So, but normal things that everybody gets, you usually go, ‘Oh, okay, take some Tylenol’. But then I worry about bad... not bad things, but things come to your mind that don’t normally for a healthy person.”

[P14]

#### 3.2.3. Self-Silencing

The participants mentioned that they were withholding their FCR from the patient because they did not “want [the patient] to start worrying”. They expressed a strong desire to protect their loved ones from further pain by hiding their own. Despite the participants experiencing heavy and hard-to-handle emotions, they chose to prioritize the patient’s needs over their own. Although patients expressed a desire to know about the participants’ feelings, the participants decided to withhold sharing the truth to protect the patient, as follows:

“I get worried sick, confused, sad and devastated. Whenever she tries to find out about what’s wrong, I keep declining to tell her just so she won’t feel like she’s been a burden to me.”

[FGP5]

#### 3.2.4. FCR Is Isolating

A poignant aspect of the participants’ experience with FCR was their isolation. The participants repeatedly described being “lonely” and “alone”, because despite having friends and family, “nobody [understood their] situation”. This led to significant distress, as follows:

“I felt very lonely, very alone. And I just, I kept reaching out and nobody… I didn’t know, I was in such, um, distress with my daughter. We didn’t know who to call. We didn’t know what to do. We just, we were alone. We were alone all the time and struggling.”

[FGP1]

Moreover, the participants were unable to find “like-minded” people who could relate to the “trauma” they faced and their FCR. Thus, the caregivers were led to question the normalcy of their fear, as follows:

“It’s really hard to explain what I was... I think I was feeling very isolated in my experience upon reflection, for example, I didn’t know whether my feelings were at all normal.”

[P7]

Additionally, some participants could not be sufficiently supported by those around them, whether due to a lack of understanding or challenges in receiving support from other family members. This caused them to feel an immense burden of handling their FCR alone, as follows:

“I used to talk to my brother… And I don’t talk to him about it anymore because, it’s that thing of people have reached their saturation point. It’s been two years now. People don’t [want to] keep hearing about it, right? For them, it’s off their radar. Whereas I’m still in the middle of it, and I don’t [want to] be this doom and gloom… And I can almost, even though we’re on the phone, I can almost see his eyes kind of glaze over. He doesn’t want to hear it anymore. And I get it. I truly get it. So, I just don’t bring it to him anymore.”

[FGP2]

#### 3.2.5. Finding Support

Faced with overwhelming FCR, the participants discussed their approach to finding resources and support. Several participants brought attention to the lack of available resources for family caregivers in favour of providing them to patients. The participants felt that “[their] needs are second to the person who’s ill” and that “[programs] don’t even think about [FC], that [they] might need something”. One participant described it as follows:

“Like, he’s in treatment. He’s going through all that. There’s not that many resources for me or any other caregiver, you’re very much beside the system.”

[P8]

The participants who had accessed external support from peer support groups spoke about finding others who they could “relate to” and gaining “comfort” in both sharing and listening to their peers’ experiences with FCR, as follows:

“See if the way that I feel, if I’m overreacting… So, I just wanted to know if I was over the top or in the same boat as other caregivers… Like I said before, I got family and friends and they’re all sympathetic and, you know, I know they all care. But they don’t know my feelings and the group, they everybody was saying how they felt and how the fear of recurrence impacts them, and I could relate to that.”

[P11]

“I also think having the other group members there. I gained so much comfort from having a space where people talked about this freely because it really in your day-to-day life, everyone around me is aware of what’s going on, but I would never just openly or comfortably talk about [it] with very few of them how scary it is and expect them to understand. Verses this space, like everyone really did understand and was dealing with it in their own way.”

[P8]

Other participants mentioned how peer support was a source of empowerment, companionship, and inspiration for helpful fear management techniques, as follows:

“I think even listening to the other women and their fears and coping techniques and what they do to recharge their battery, it brings the tone that we’re worthy and “oh I never thought of doing that to recharge my battery, that’s quicker” and “oh I think I’ll try that.” Kind of made an impact.”

[P3]

Similarly, professional support, including that from physicians, counsellors, and psychologists, was perceived to be “compassionate” and taught them how to “deal with [FCR]”. For some participants, faith and spirituality were important external guiding factors, and they would “reach out to people to pray for [the patient] and to pray for [the participant]”.

## 4. Discussion

This qualitative study used a focus group and semi-structured interviews to explore the experiences of family caregivers in regard to FCR and identify their unique supportive needs. Through reflexive thematic analysis [25], we uncovered details about family caregivers’ experiences of FCR. Most notably, FCR was experienced as persistent and all-consuming and was shaped by relational factors. A primary theme centered around the relational aspects of FCR in family caregivers was identified, including the following four accompanying sub-themes: patient-centric hypervigilance, self-silencing, FCR as isolating, and finding support. Our findings are consistent with the previous literature [19,23,24] emphasizing the distinct aspects of FCR experiences in family caregivers. However, our findings also present a unique perspective on the relational aspects of FCR and the supportive needs of family caregivers.

The family caregivers reported high levels of fears and worries related to the possibility of recurrence. They described their FCR as constant, all-consuming, and triggered by various factors. Importantly, they reported engaging in excessive hypervigilance of their patients’ well-being. Similar to previous studies on the understanding of FCR in patients [12] and family caregivers [19], symptomology represented a key trigger for family caregivers. Our findings also reflect those of Webb [6,19,23] and Banks [24], which indicate that family caregivers experience high levels of fear, uncertainty, triggers (primarily symptomology), and hypervigilance related to FCR.

The family caregivers reported a strong desire to protect their loved ones from their fears and prioritizing the patient’s needs before theirs. As a result, they described self-silencing their worries by avoiding sharing their thoughts and feelings with patients or avoiding thinking about their FCR. Protective buffering, or the act of “withholding or denying cancer-related thoughts and concerns from one’s partner, hiding dispiriting information, and acquiescing to avoid conflict” [31], is associated with poorer psychological outcomes [31], decreases in global relationship quality and intimacy [32], and increases in FCR for both patients and family caregivers [32,33]. Therefore, addressing protective buffering can be beneficial for both caregivers and patients, and FCR interventions designed for family caregivers should include targeted exercises aimed at helping them engage in conversations about FCR.

Moreover, a key finding from this study is the significant isolation experienced by family caregivers—often despite having good support systems. Our results align with the existing literature, suggesting that cancer family caregivers typically report moderate to high levels of loneliness [34]. Loneliness has been associated with negative consequences on physical (e.g., sleep, blood pressure, lifestyle, inflammation, and mortality) and psychological (e.g., anxiety, depression, fatigue, and suicidality) health [34]. Furthermore, factors such as marital and employment status, the presence of mental health difficulties, increased stress, and caregiving burden have been linked to increases in loneliness in family caregivers [35]. Our findings suggest the importance of group interventions for family caregivers to re-establish social connection and support.

Our results also propose that interventions focusing on the sharing of experiences between family caregivers or with healthcare professionals represent more suitable options. In fact, the participants primarily described seeking external sources of support (peer, professional, and spiritual) to help them navigate their FCR. Peer and spiritual support represented an important source of comfort, empowerment, and relatedness for family caregivers. Professional support provided a space for family caregivers to learn how to manage their FCR. Similarly to Webb [23], almost all participants expressed being unsupported in their caregiver role and being unable to access appropriate support. Most notably, they described difficulties in sharing their experiences with their inner circle, as they felt that others did not understand or could not relate to their situation.

Finally, the family caregivers highlighted that their needs were often considered as secondary to those of the patients, which resulted in a lack of available formal and informational support received. Our results further contribute to the growing importance of healthcare providers having the ability to appropriately assess and identify FCR-related distress in family caregivers. Measures for the assessment of FCR in family caregivers, such as the caregiver version of the Fear of Cancer Recurrence Inventory [30] and the Caregiver Fear of Cancer Recurrence/Progression Measure (CARE-FCR) [36], are beginning to emerge. However, more research is needed to generate an agreed upon and criteria-specific model of FCR in family caregivers. Furthermore, more research is needed to evaluate the validity and reliability of family caregiver measures of FCR and include them in clinical care. While some interventions [14] address cancer-related distress in family caregivers or family caregiver–patient dyads, to our knowledge, only the FC-FORT [26,27,28] intervention currently exists to specifically target FCR in family caregivers. Given the impact of FCR on family caregivers, and subsequently survivors, further research should be invested in the development and implementation of family-caregiver-specific interventions in healthcare contexts.

Overall, while similarities between patients’ and family caregivers’ experiences of FCR exist, our findings propose a key difference in the mechanisms that influence family caregivers’ FCR. In fact, it appears that, for family caregivers, relational aspects (patient-centric hypervigilance, self-silencing, isolation, and lack of available support) significantly increase and impact their experience of FCR.

### 4.1. Study Limitations

Due to the small size and homogeneity of our sample, we may have missed critical information regarding family caregivers’ experiences of FCR. Most family caregivers were White women, and all were English-speaking Canadians, which challenges the transferability of our results. Further research should investigate the distinct aspects of FCR among more culturally diverse communities of family caregivers. Additionally, other limitations include the potential for digital exclusion given the virtual nature of the focus group and interviews, as well as the combination of data sets from related, but separate studies.

### 4.2. Clinical Implications

While research on FCR in family caregivers is rapidly growing, we still have a limited understanding of the experiences of family caregivers living with FCR. Previous research has established that, while similarities exist between patients’ and family caregivers’ experiences of FCR (e.g., consistency, uncertainty, triggers, and hypervigilance), the driving factors differ from those of patients (i.e., sense of responsibility for patient health and fear of loved one dying). Our results echo those of previous research while highlighting key aspects of the management of FCR in family caregivers. Namely, important findings such as the impacts of family caregivers’ isolation, lack of access to available resources, and the importance of external support (i.e., outside of social support) are crucial to understanding the FCR experience in family caregivers. As a result, assessments of FCR in family caregivers should also include considerations related to support, as this may impact the severity of symptoms. Finally, clinicians and researchers should strongly consider the importance of incorporating relational aspects into the development of interventions designed to reduce FCR in family caregivers.

## 5. Conclusions

Our study explored the experience of family caregivers living with FCR and their unique needs. Key themes related to the experience of FCR, the patient–caregiver relationship, and family caregivers’ management of FCR were identified. Our results suggest that FCs’ experiences of FCR mirror, in part, the experiences of patients, however, notable differences were also identified. In addition, the results suggest that family caregivers would benefit from access to psychosocial services, which are currently lacking, and specifically external sources of support.

## Figures and Tables

**Table 1:** Sample characteristics.

	Total (*n* = 20)
**Gender (Women)**	19 (95%)
**Age, M (SD) (Range)**	51.4 (15.99) (21–74)
**Race (%)**	
White	13 (65%)
South Asian	2 (10%)
Chinese	1 (5%)
Hispanic	1 (5%)
Unknown	3 (15%)
**Marital Status (%)**	
Single, Never Married	2 (10%)
Married/Common Law Partners	13 (65%)
Separated, Divorced	1 (5%)
Unknown	4 (20%)
**Education (%)**	
Part of University/College	4 (20%)
University/College	11 (55%)
Graduate School	4 (20%)
Professional Program	1 (5%)
**Occupational Status (%)**	
Employed Full-Time	8 (40%)
Employed Part-Time	5 (25%)
Retired	4 (20%)
Student	1 (5%)
Unemployed	2 (10%)
**Annual Income (%)**	
CAD 21,000–40,000	1 (5%)
CAD 41,000–60,000	4 (20%)
CAD 61,000–80,000	3 (15%)
CAD 81,000–100,000	2 (10%)
Greater than CAD 100,000	5 (25%)
Unknown	5 (25%)

**Table 2:** Patient characteristics.

Family Member’s Cancer Type (%)	
Breast	4 (20%)
Prostate	4 (20%)
Hodgkin’s Lymphoma	3 (15%)
Leukemia	3 (15%)
Ovarian	1 (5%)
Kidney	1 (5%)
Glioblastoma Multiforme	1 (5%)
Melanoma	1 (5%)
Rhabdomyosarcoma of Uterine Cervix	1 (5%)
Pancreatic	1 (5%)
**Family Member’s Cancer Stage (%)**	
I	2 (10%)
II	3 (15%)
III	4 (20%)
IV	2 (10%)
Unknown/Other	9 (45%)
**Caregiver Relationship with Family Member (%)**	
Spouse/Partner	9 (45%)
Parent	5 (25%)
Child	5 (25%)
Other (e.g., Aunt)	1 (5%)

## Data Availability

Please contact the corresponding author regarding any data requests.

## Abbreviations

The following abbreviations are used in this manuscript:

FCR	Fear of Cancer Recurrence
FORT	Fear Of Recurrence Therapy
FC-FORT	Family Caregiver-Fear Of Recurrence Therapy
QoL	Quality of Life
